# Impact of Maternal Selenium Status on Infant Outcome during the First 6 Months of Life

**DOI:** 10.3390/nu9050486

**Published:** 2017-05-11

**Authors:** Kristin Varsi, Bjørn Bolann, Ingrid Torsvik, Tina Constanse Rosvold Eik, Paul Johan Høl, Anne-Lise Bjørke-Monsen

**Affiliations:** 1 Laboratory of Clinical Biochemistry, Haukeland University Hospital, N-5021 Bergen, Norway; bjorn.bolann@uib.no (B.B.); tina.constanse.rosvold.eik@helse-bergen.no (T.C.R.E.); almo@helse-bergen.no (A.-L.B.-M.); 2 Department of Clinical Science, Faculty of Medicine and Dentistry, University of Bergen, N-5020 Bergen, Norway; 3 Department of Pediatrics, Haukeland University Hospital, N-5021 Bergen, Norway; ingrid.kristin.torsvik@helse-bergen.no; 4 Department of Clinical Medicine, Faculty of Medicine and Dentistry, University of Bergen, N-5020 Bergen, Norway; paul.hol@k1.uib.no

**Keywords:** selenium, deficiency, infant, pregnancy, lactation, infection, neurodevelopment

## Abstract

Pregnant women and infants are at risk for selenium deficiency, which is known to have negative effects on immune and brain function. We have investigated selenium levels in 158 healthy never-pregnant women and in 114 pregnant and lactating women and their infants at age 6 months and related this to clinical outcomes during the first 6 months of life. Neurodevelopment was assessed with the parental questionnaire Ages and Stages (ASQ) at 6 months. A maternal selenium level ≤0.90 µmol/L in pregnancy week 18 was negatively related to infant neurodevelopment at 6 months (B = −20, *p* = 0.01), whereas a selenium level ≤0.78 µmol/L in pregnancy week 36 was associated with an increased risk (odds ratio 4.8) of having an infant infection during the first 6 weeks of life. A low maternal selenium status in pregnancy was found to be associated with an increased risk of infant infection during the first 6 weeks of life and a lower psychomotor score at 6 months. We suggest a cutoff for maternal serum selenium deficiency of 0.90 µmol/L in pregnancy week 18 and 0.78 µmol/L in pregnancy week 36. This should be reevaluated in an intervention study.

## 1. Introduction

Selenium is a trace element essential for normal human metabolism, and even less overt selenium deficiency is reported to have negative health effects in humans [1]. Overt deficiency is typically associated with loss of immunocompetence, affecting both cell-mediated and humoral immune function [1,2], and selenium supplementation has been shown to improve immune function even in selenium replete individuals [3].

A low maternal selenium status during pregnancy has been associated with fetal malformations, like neural tube defects [4], and is disadvantageous for cognitive development in infants and toddlers [5,6]. However, due to extensive physiological changes during pregnancy [7], the definition of maternal selenium deficiency is difficult in this period. Serum selenium levels decrease during pregnancy with the lowest levels observed before delivery [6,8,9], and increase postpartum to levels observed in non-pregnant women [8]. Selenium levels in breastmilk depend on maternal selenium status, and are reported to decrease during the lactational period [10]. It has been estimated that recommended selenium intake is not achieved in approximately 30% of breastfed infants [10], and infants are considered to be particularly at risk for selenium deficiency [11]. Serum selenium decreases from birth to reach nadir levels between 2 to 4 months, increases thereafter and is higher in older children [11,12,13]. 

Seafood and meat are rich sources of selenium, whereas the selenium content in plant-based foods depends on where it is grown. As the selenium content in soil and food habits vary substantially in different world regions, this will affect the selenium status in different populations [1,14]. Northern Europe represents a low selenium area [1], and low selenium levels have been reported in inhabitants from the Nordic countries [14,15]. 

We have investigated selenium levels in Norwegian never-pregnant and pregnant women during pregnancy and postpartum, in breastmilk, and in their infants at age 6 months. Statistically based reference ranges may not be optimal for securing an adequate micronutrient level in pregnant women and their infants. Our purpose was to establish cut-off levels for maternal selenium deficiency during pregnancy based on clinical outcomes in the infants during the first 6 months of life.

## 2. Materials and Methods

### 2.1. Study Population and Design

Between June 2012 and March 2015, 140 healthy pregnant women with a singleton pregnancy were recruited at routine ultrasound examinations in pregnancy week (PW) 18 at the Obstetrical Department at Haukeland University Hospital, Bergen, Norway. The women were invited back at pregnancy weeks 28 and 36, 6 weeks and 4 and 6 months postpartum, and the final visit also included the infant. Women with pregnancy related or chronic disease were excluded, except those with well-regulated hypothyroidism (*n* = 7). Of the 140 pregnant women initially recruited, 114 met the inclusion criteria, and were included in the study.

During the same period, 158 healthy, never-pregnant women aged 18 to 40 years were recruited among students and employees at the University of Bergen and Haukeland University Hospital, Bergen, Norway. 

Ethical approval of the protocol was granted by the Regional Committee on Medical Research Ethics, REK 2011/2447, and written informed consent was obtained from all women before enrollment. 

### 2.2. Clinical Data

The subjects completed a questionnaire concerning body weight, nutrition, use of multiple micronutrient supplements (MMN), and health status at each visit. The postpartum visits included additional information about infant nutrition, growth parameters, and clinical symptoms concerning feeding difficulties, colic, constipation, dermatitis, and infections. Infections were defined as clinical symptoms compatible with infection with or without fever. Fever-episodes occurring after scheduled vaccinations were excluded. Use of MMN more than three days per week in two or more periods during pregnancy or postpartum period was defined as a regular supplement user.

Infant neurodevelopment was assessed by Ages and Stages Questionnaire: A Parent-Completed, Child-Monitoring System (ASQ), a screening tool that includes five developmental domains: communication, gross motor function, fine motor function, social functioning, and problem solving [16]. Each domain had six questions, and the parents assessed whether a milestone was achieved (yes, 10 points), partially achieved (sometimes, 5 points), or not achieved (no, 0 points). Partial scores of each domain and a total score (maximum 300 points) were calculated for each infant. 

### 2.3. Selenium Analysis of Blood and Breastmilk

Non-fasting blood samples from women and infants were obtained by antecubital venipuncture and collected into vacutainer tubes without additives and approved for trace metal analysis (Terumo, Tokyo, Japan). A complete set of blood samples was available for all, except nine women and 16 infants. Selenium in serum was measured by Inductively Coupled Plasma Mass Spectrometry (ICP-MS) on Elan^®^ Dynamic Reaction Cell-e (Perkin Elmer MDS Sciex, Concord, ON, Canada) in standard mode [17]. The serum samples were diluted 1:15 with dilution solution containing 1% Triton^®^ X-100 (Merck, Damstadt, Germany) 0.33% w/v pro analysis HNO_3_ (Merck, Germany), and 5 ppm Gold (Perkin Elmer, Shelton, CT, USA). Seronorm Trace Elements serum Level 1 and 2 (Sero, Billingstad, Norway) were included in every analysis series. The analytical between run precision within the normal range was <4%. Twenty-two measurements of a low serum pool with a concentration of 0.39 μmol/L gave a coefficient of variation (CV) of 5.4 %, and this concentration was set as the limit of quantification (LOQ). No samples were below the LOQ. The average recovery for the serum samples was 99%.

At each postpartum visit, the mothers (*n* = 61) brought a sample of breastmilk taken the same day and stored in tubes without additives, and approved for trace metal analysis. Breastmilk samples were thawed and 1 mL was mixed with 2 mL ultrapure HNO_3_, 65%, and 1 mL ultrapure H_2_O_2_, 30%, before microwave digestion (MLS 1200 Mega, Milestone, Sorisole, Italy). Thereafter, the samples were mixed with 0.25 mL suprapure HCl, 30%, before analysis by high resolution sector field ICP-MS on Element 2 (Thermo Finnigan, Bremen, Germany) [18]. For quality control, a Seronorm Trace Elements serum Level 2 (Sero, Billingstad, Norway) was used. One mL of serum followed the sample preparation as the breastmilk samples. In addition, a reference material of skimmed milk powder (ERM-BD-150, Geel, Belgium) was used. The analytical precision between runs was 4% for the serum control and 12% for the milk powder. For the breastmilk method, the standard deviation (SD) of the blank was 0.017 μmol/L. This gives a theoretical limit of detection (LOD) (defined as three times the SD of the blank) of 0.052 μmol/L. The average recovery for the serum and milk powder was 109% and 106%, respectively, indicating that the selenium concentration may have been slightly overestimated. 

### 2.4. Statistical Analysis

Normally distributed data are presented as means and SD, and compared by Student’s *t*-test, whereas non-normally distributed data are presented as medians and interquartile ranges (IQR) defined by 25 and 75 percentiles, or 2.5 and 97.5 percentiles, and compared by Mann-Whitney U test or Kruskal Wallis test. Categorical data are presented as percentages compared by Chi-square test. Spearman correlations were used to explore relationships between data. Selenium tertiles were used in linear or logistic regression models due to a small sample size and more interest in extreme quantiles than in the mean values. Multiple linear regression models were used to assess the relationship between ASQ scores at 6 months and maternal selenium status during pregnancy. The unstandardized coefficient (B) represents an estimate of the standardized β coefficient, and the models also included variables chosen due to their reported impact for neurodevelopment [19]. Logistic regressions models, including factors considered to be related to the risk of infection during the first weeks of life, were used to assess the risk of having an infant infection during the first 6 weeks of life in relation to maternal selenium levels during pregnancy and postpartum. The SPSS statistical package (version 23) was used, and two-sided *p*-values < 0.05 were considered statistically significant.

## 3. Results

### 3.1. Demographics and Nutrition

All of the mothers had an omnivore diet, and the majority used multiple micronutrient supplements (MMN) containing a low dose selenium (55–60 µg/tablet) at more than 2 periods during pregnancy (86/114, 75%) and postpartum (82/114, 72%). 

Compared to the mothers, the never-pregnant controls (*n* = 158) were younger, 23% (39/158) were vegetarians, and a lower percentage were regular MMN users (Table 1). 

All infants (*n* = 114) were healthy, 53% (60/114) were males, and all infants except one (born at gestational age 36 weeks) were born at term, with an appropriate for gestational age weight (mean 3573 + 418 g). All, except one infant, were breastfed, with a mean duration of exclusive breastfeeding of 3.8 + 1.5 months. At four months, 50% (70/114) of the infants had been introduced to solid food. Use of vitamin D supplements was reported in 60% of the infants at 6 weeks, increasing up to 90% at 6 months, and no infant was given MMNs or iron supplements at any time point. 

### 3.2. Selenium Levels in Pregnant, Lactating, and Never-Pregnant Women 

**Figure 1:**
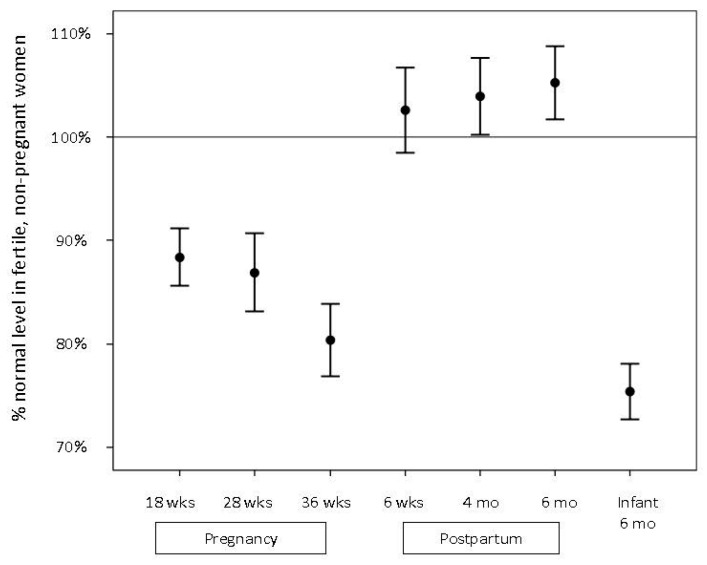
Selenium levels in pregnant and lactating women and their infants, expressed as the percentage of median selenium level in never-pregnant women (mean + 2 SD).

Serum selenium decreased significantly during pregnancy (*p* < 0.001), and the median level in pregnancy week 36 was reduced by 20% compared to never-pregnant women (*p* < 0.001) (Figure 1). The levels increased postpartum and remained unchanged from 6 weeks to 6 months where they were comparable to never-pregnant levels (Table 2, Figure 1). Spearman rank correlation coefficients (*r*) between maternal serum selenium levels during pregnancy and postpartum were all >0.5, *p* < 0.001, and maternal selenium levels were higher in regular users of MMN (Table 3).

No significant correlations were seen between serum selenium and body mass index (BMI), age, or the use of MMN in the never-pregnant controls. 

### 3.3. Selenium Levels in Breastmilk

Breastmilk selenium levels at 6 weeks and 4 and 6 months were significantly correlated to maternal selenium levels both during pregnancy and postpartum, with the highest correlations seen for maternal postpartum levels (*r*: 0.28–0.55, *p* < 0.03). No significant differences in breastmilk selenium levels according to maternal use of MMN were observed (*p* > 0.22). The median selenium level decreased by 23% between 6 weeks and 4 months (*p* < 0.001), with no change from 4 to 6 months (*p* = 0.90) (Table 4). 

### 3.4. Selenium Levels in Infants at 6 Months

Infant selenium levels at age 6 months were lower than in never-pregnant, pregnant, and lactating mothers (Table 2), correlated to maternal levels during pregnancy and postpartum (*r* = 0.28–0.45, *p* < 0.01), and were higher in infants of mothers who were regular users of MMN during pregnancy (*p* = 0.05) and postpartum (*p* = 0.03) (Table 3). 

Calculated median selenium intake from breastmilk decreased from 6 weeks to 6 months (*p* = 0.02) (Table 4), but the median selenium level in the infants at 6 months did not significantly differ according to months of exclusive breastfeeding, neither to gender or growth parameters during the first 6 months of life. 

### 3.5. Selenium Levels and Infant Neurodevelopment

ASQ data were available for 98% (112/114) of the infants at 6 months. Median and IQR were 228 (210, 255) for total ASQ score, 50 (40, 55) for communication score, 50 (39, 55) for personal-social functioning score, 55 (46, 60) for problem solving score, 35 (30, 45) for gross motor score, and 45 (35, 55) for fine motor score. 

In a multiple linear regression model, ASQ total (B = 11, *p* = 0.007), problem solving (B = 3, *p* = 0.006), and fine motor scores (B = 3, *p* = 0.04) increased significantly with maternal selenium tertiles in pregnancy week 18, whereas communication (B = 1, *p* = 0.22), personal-social functioning (B = 2, *p* = 0.11), and gross motor score (B = 1, *p* = 0.56) did not. The multiple linear regression model also included birthweight, weight at 6 months, gender, months of exclusive breastfeeding, maternal age, education, and parity. 

Based on examination of scatterplots visualizing the relationship between maternal serum selenium in pregnancy week 18 and infant ASQ scores at 6 months, the lower tertile of maternal serum selenium (<0.90 µmol/L) was chosen as a cutoff level for defining maternal selenium deficiency in pregnancy week 18. Infants born to mothers with serum selenium <0.90 µmol/L in pregnancy week 18 (lower tertile) had significantly lower total, problem solving, personal-social functioning, and fine motor function ASQ scores compared to infants born to mothers with higher selenium levels (Table 5, Supplemental Figure S1).

No significant associations were observed between ASQ scores and maternal selenium status at other timepoints or for infant selenium status (data not shown). 

### 3.6. Selenium Levels and Infant Infections

The prevalence of reported infant infections increased during the first 6 months of life from 18% (19/107) during the first 6 weeks to 35% (39/113) from 6 weeks to 4 months and to 44% (50/114) from 4 to 6 months. Of the infections in the first 6 weeks of life (sepsis (*n* = 1), lower airway infection (*n* = 2), upper airway infection (*n* = 8), lower urinary tract infection (*n* = 1), dermal or mucosal infections (*n* = 8)), 32% (6/19) were associated with fever and 26% (5/19) required antibiotics. Maternal serum selenium levels in pregnancy week 36 and 6 weeks postpartum were significantly lower in mothers of infants who had a reported infection during the first 6 weeks of life, compared to mothers of healthy infants (Table 6). 

Based on examination of scatterplots, box-plot and error bars visualizing the relation between maternal serum selenium in pregnancy week 36 and the occurrence of infant infection during the first 6 weeks of life, the lower tertile of maternal serum selenium (<0.78 µmol/L) was chosen as a cutoff level for defining maternal selenium deficiency in pregnancy week 36. The infants had an increased risk with an odds ratio (OR) of 4.8 (95% CI 1.2–20.5) for having an infection during the first 6 weeks of life if the maternal serum selenium level was in the lower tertile (≤0.78 µmol/L) in pregnancy week 36, and an OR of 2.5 (95% 0.7–9.5) if maternal selenium was in the lower tertile (<0.99 µmol/L) at 6 weeks postpartum. These results are from a logistic regression model which additionally included birthweight, weight at 6 weeks, gender, weeks of exclusive breastfeeding, parity, and incidence of maternal infections during the same period (Supplemental Figure S2).

No significant relationships were observed between maternal selenium status during pregnancy and lactation and the occurrence of maternal infections postpartum. We did not observe any significant correlations between maternal and infant selenium status to any other reported condition (feeding difficulties, colic, constipation, or dermatitis) in the infant during the first 6 months of life.

## 4. Discussion

Compared to never-pregnant women, median maternal selenium levels were reduced by 20% in later pregnancy, increased postpartum, and remained unchanged for the first 6 months. Maternal selenium levels in pregnancy and postpartum were highly correlated to breastmilk and infant selenium levels. A maternal selenium level in the lower tertile in pregnancy week 18 (≤0.90 µmol/L) was negatively related to infant neurodevelopment at 6 months, whereas a selenium level in the lower tertile in pregnancy week 36 (≤0.78 µmol/L) was associated with an increased risk (OR 4.8) of infant infection during the first 6 weeks of life.

### 4.1. Serum Selenium Levels in Never-Pregnant, Pregnant, and Lactating Women 

In adults, a plateau in plasma selenoprotein P has been reached with serum selenium concentrations >1.57 µmol/L [20]; this is considered to indicate a selenium-replete status [21]. Median serum selenium levels are reported to be lower in females compared to men (1.18 vs. 1.27 µmol/L), lower in subjects younger than 40 years compared to older subjects (1.08 vs. 1.24 µmol/L), and lower in adult Norwegians compared to adult Americans (1.20 vs. 1.51 µmol/L) [14,21]. In our population of healthy never-pregnant women, the median level was 1.07 µmol/L and 98% (154/158) had a selenium level below 1.57 µmol/L, indicating a non-replete selenium status. 

Median selenium levels in our pregnant population varied from 0.96 µmol/L in week 18 to 0.85 µmol/L in week 36, comparable to reported selenium levels in pregnancy week 15–22 ranging from 0.54 µmol/L in Polish women [6], to 1.25 µmol/L in Spanish women [9], with lower levels observed just before delivery [6,8,9]. The levels increased postpartum, and were higher in regular users of MMN throughout pregnancy and postpartum, as has been observed by others [8], however, almost all (97–99%) values were below 1.57 µmol/L at all time points. 

### 4.2. Selenium Levels in Breastmilk

Milk selenium level is closely related to maternal selenium status and varies substantially between world regions and with time after birth [10]. Median selenium levels are reported to be 26 µg/L (0.33 µmol/L) in colostrum (0–5 days), decrease to 18 µg/L (0.23 µmol/L) in transitional milk (6–21 days), decrease further to nadir levels of 15 µg/L (0.19 µmol/L) in mature milk (1–3 months) and be slightly higher 17 μg/L (0.22 µmol/L) in late milk (>5 months) [10]. Compared to these data, selenium levels in breastmilk from mothers in Northern Europe is reported to be lower [10], as demonstrated by our data. 

### 4.3. Selenium Intake and Serum Levels in Infants 

The recommended daily selenium intake up to 4 months is set to 10 µg (0.13 µmol) and based on the assumption that selenium levels in breastmilk are optimal for this age-group [22]. For infants 4 to 12 months, the recommended daily intake is 15 µg (0.19 µmol), which is still based on selenium milk values and estimated increase in average body weight as no data is available on selenium intake from solid food in infants [22]. In our population, median selenium intake from breastmilk decreased during the lactational period and was 77% of the recommended level at 6 weeks, and 69% at 6 months, which is comparable to published data in breastfed Polish infants, in whom both plasma selenium and glutathione peroxidase-3 levels increased after maternal selenium supplementation, indicating a former selenium deficiency [23]. 

Infants are considered to be at risk for selenium deficiency, and the levels are reported to decrease during the first months of life [11]. In German children, median serum selenium decreased from 0.64 µmol/L in infants less than 1 month to 0.44 µmol/L at 4 months, increased to 0.62 µmol/L between 4 to 12 months, remained stable between 1 to 5 years (0.90 µmol/L), and increased slightly from 5 to 18 years (median 0.99 µmol/L) [11]. 

A pattern with nadir serum levels in infants between 3 to 6 months is observed also for other micronutrients, like cobalamin and iron, and is associated with a negative clinical outcome [24,25,26], merely reflecting the vulnerable nutrient status during the first months of life. The median selenium level in Norwegian infants at 6 months (0.81 µmol/L) was within the established reference range based on German children [11], however, reference ranges may not be an optimal method for evaluating micronutrient status, particularly when based on populations with a high prevalence of deficiency.

### 4.4. Selenium Levels and Infant Clinical Status

The obtained ASQ scores in our infant population resemble published data in healthy, Norwegian infants at 6 months [27]. A study from Poland reported a positive association between maternal selenium levels in the first trimester (mean 0.61 (SD 0.13) µmol/L) and motor development at 1 year of age, and language development at 2 years [6]. Similar associations between maternal selenium status and neurodevelopment have been reported in other human [5] and animal studies [28]. Our observed lower ASQ scores (total, problem solving, personal-social functioning, and fine motor function) in infants born to mothers with a selenium level ≤0.90 µmol/L in pregnancy week 18 are in line with these data. 

Neurodevelopment is multifaceted, and factors like birthweight, weight increase after birth, gender, breastfeeding, maternal age, education, and parity are reported to have an impact on neurodevelopment [19]. However, ASQ total score, problem solving, personal-social functioning, and fine motor score still increased significantly with maternal selenium tertiles in pregnancy week 18, even when such factors were included in the multiple linear regression models. Neurodevelopment is also dependent on a range of nutritional factors, and we cannot exclude that nutrients other than selenium may have contributed to the observed differences in clinical outcome. Further, it is likely that women with an adequate selenium status also have an adequate status for other micronutrients due to a healthy diet or MMN supplements.

We also observed significantly lower maternal selenium levels in pregnancy week 36 and 6 weeks postpartum in mothers of infants who had an early infection. A logistic regression model, which additionally included factors considered to be related to the risk of infection, demonstrated a significantly increased risk (OR 4.8) of infant infection during the first 6 weeks of life if the maternal serum selenium level in pregnancy week 36 was ≤0.78 µmol/L. Studies have evaluated clinical infant outcomes related to selenium status in HIV-infected pregnant women [29], but apart from this, data on the association between maternal selenium status in pregnancy and infant immunocompetence has, to our knowledge, not been previously published. 

While maternal selenium status in pregnancy week 18 was related to neurodevelopment, maternal status in the last trimester was related to risk of infection. Brain development begins in the third gestational week and continues after birth [30]. The preventive effect of folic acid supplementation on neural tube defects [31] has demonstrated the importance of an adequate micronutrient status in early pregnancy for normal neurodevelopment, and our findings may justify a role for selenium in early neurodevelopment as well. 

Fetal micronutrient stores are formed during the last trimester, and a low maternal selenium status during this period will reduce fetal stores. A low selenium status may negatively influence both the humoral and cell-mediated immune function [32], and maternal antenatal and postpartum selenium supplementation have been associated with a reduced child mortality after 6 weeks of age [29]. 

### 4.5. Strength and Limitations

This was an observational study with a small sample size of mother and infant dyads, and the clinical data were reported by the mothers, factors known to be disadvantageous. The data were, however, collected prospectively throughout pregnancy and postpartum and the participation rate was high, which are strengths to the study. Evaluation of neurodevelopment in young infants is challenging [33], but ASQ is a validated screening tool with high sensitivity and specificity to detect children with developmental delay [16]. The maternal reported infant infections included diagnoses defined by quite distinct and well-known symptoms of infection, unlikely to be confused by other diagnoses, like allergy. 

## 5. Conclusions

An adequate selenium status is important for fetal and infant development. It is, therefore, important to optimize maternal selenium status during pregnancy. As the interpretation of maternal selenium status is hampered in pregnancy, due to numerous physiological changes, we suggest to use clinical infant outcome in order to establish selenium cut off levels in pregnancy. In a Norwegian population of healthy pregnant women, a low maternal selenium status was associated with a lower psychomotor score at 6 months and an increased risk of infant infection during the first 6 weeks of life. Based on our observations, we suggest a cutoff for maternal serum selenium deficiency of 0.90 µmol/L in pregnancy week 18, and 0.78 µmol/L in pregnancy week 36. This should be evaluated in a randomized intervention study. 

## Figures and Tables

**Table 1:** Baseline characteristics of never-pregnant and pregnant women in pregnancy week 18. BMI = body mass index.

	Never-Pregnant Women	Pregnant Women	*p* Value
	*n* = 158	*n* = 114	
Age, year, mean + SD	25.3 + 4.8	31.5 + 4.3	<0.001 *
Prepregnancy BMI, kg/m^2^, mean + SD	22.5 + 3.0	22.8 + 3.1	0.38 *
Higher education, *n* (%) **	93 (60)	67 (59)	0.96 ***
Para 0, *n* (%)	158 (100)	63 (55)	<0.001 ***
Smoking, *n* (%)	4 (3)	2 (2)	0.65 ***
Regular use of micronutrient supplements (≥3 days/week), *n* (%)	35 (22)	47 (41)	0.001 ***

* Comparison by Student’s *t*-test; ** Higher education defined as ≥5 years at university or college, *** Comparison by Pearson Chi-square test.

**Table 2:** Serum selenium levels in never-pregnant women, women during pregnancy and postpartum, and infants at 6 months.

Serum Selenium, µmol/L	Never-Pregnant Women *n* = 158	Pregnant Women			Postpartum Women			Infants 6 Months *n* = 91
		Week 18 *n* = 108	Week 28 *n* = 114	Week 36 *n* = 114	6 Weeks *n* = 113	4 Months *n* = 113	6 Months *n* = 114	
Median	1.07	0.96	0.92	0.85	1.07	1.08	1.11	0.81
(25, 75) *	(0.98, 1.16)	(0.86, 1.04)	(0.81, 1.03)	(0.75, 0.96)	(0.96, 1.20)	(0.98, 1.24)	(1.00, 1.25)	(0.74, 0.89)
(2.5, 97.5) *	(0.81, 1.59)	(0.71, 1.35)	(0.67, 1.37)	(0.63, 1.33)	(0.74, 1.58)	(0.86, 1.59)	(0.88, 1.51)	(0.56, 1.16)

* Percentiles.

**Table 3:** Maternal and infant serum selenium according to maternal use of micronutrients in pregnancy and postpartum.

Use of Micronutrient Supplements during Pregnancy	Maternal Serum Selenium, µmol/L, Median (25, 75) * (*n* = 114)			Infant Serum Selenium, µmol/L, At Age 6 Months, Median (25, 75) * (*n* = 91)	
	Pregnancy 18 Weeks	Pregnancy Week 28	Pregnancy Week 36		
Non-user, *n* = 28	0.92 (0.83, 1.00)	0.83 (0.77, 0.98)	0.77 (0.73, 0.86)	*n* = 25	0.76 (0.71, 0.87)
Regular user, *n* = 86	0.98 (0.89, 1.05)	0.98 (0.89, 1.04)	0.87 (0.77, 0.98)	*n* = 66	0.83 (0.76, 0.90)
*p* value **	0.06	0.02	0.008	0.05	
**Use of Micronutrient Supplements Postpartum**	**6 Weeks Postpartum**	**4 Months Postpartum**	**6 Months Postpartum**	**Infants 6 Months (*n* = 91)**	
Non-user, *n* = 32	1.01 (0.95, 1.14)	1.04 (0.93, 1.14)	1.08 (0.92, 1.15)	*n* = 27	0.76 (0.69, 0.84)
Regular user, *n* = 82	1.10 (0.97, 1.27)	1.12 (0.99, 1.28)	1.12 (1.02, 1.29)	*n* = 64	0.83 (0.75, 0.90)
*p* value **	0.06	0.04	0.01	0.03	

* Percentiles, ** Comparison with Mann-Whitney U test.

**Table 4:** Selenium in breastmilk and calculated infant intake at 6 weeks and 4 and 6 months postpartum.

Parameters	6 Weeks *n* = 59	4 Months *n* = 60	6 Months *n* = 61	*p* Value *
Breastmilk selenium, µmol/L				
Median	0.13	0.10	0.09	<0.001
(25, 75) **	(0.11–0.17)	(0.08–0.13)	(0.08–0.13)	
(2.5, 97.5) **	(0.06–0.27)	(0.04–0.36)	(0.03–0.29)	
Daily selenium intake, µmol ***				0.02
Median	0.10	0.08	0.09	
(25, 75) **	(0.08, 0.12)	(0.06, 0.10)	(0.07, 0.12)	
(2.5, 97.5) **	(0.05, 0.20)	(0.03, 0.32)	(0.03, 0.29)	

* Comparison by Kruskall-Wallis test; ** Percentiles, *** Daily selenium intake was calculated based on selenium levels in breastmilk and estimated milk intake (150 mL/kg/day at 6 weeks and 120 mL/kg/day at 4 to 6 months).

**Table 5:** Ages and Stages Questionnaire (ASQ) scores for the infants at 6 months in relation to maternal serum selenium levels in pregnancy week 18.

Maternal Serum Selenium in Week 18	ASQ Scores					
	Total	Communication	Problem Solving	Personal-Social Functioning	Gross Motor Function	Fine Motor Function
<0.90 µmol/L (Tertile 1) *n* = 35	213 (189, 236)	45 (40, 50)	50 (36, 59)	45 (30, 50)	35 (30, 40)	40 (35, 50)
>0.90 µmol/L (Tertile 2–3) *n* = 70	235 (215, 258)	50 (45, 55)	55 (50, 60)	50 (40, 55)	35 (30, 45)	50 (40, 55)
*p* value *	0.002	0.12	0.005	0.02	0.57	0.02

* Compared by Mann-Whitney U test.

**Table 6:** Maternal and infant serum selenium in relation to reported infant infection from birth to age 6 weeks.

Infant Infection from Birth to Age 6 Weeks	Maternal Serum Selenium, µmol/L, Median (25, 75) *, *n* = 107				Infant Serum Selenium at 6 Months, µmol/L, Median (25, 75) * *n* = 91
	Pregnancy Week 18	Pregnancy Week 28	Pregnancy Week 36	6 Weeks Postpartum	
Yes, *n* = 19	0.99 (0.86, 1.04)	0.87 (0.80, 0.96)	0.77 (0.71, 0.86)	0.98 (0.92, 1.10)	0.77 (0.74, 0.81)
No, *n* = 88	0.96 (0.87, 1.04)	0.93 (0.81, 1.02)	0.86 (0.77, 0.96)	1.09 (0.98, 1.26)	0.83 (0.74, 0.90)
*p* value **	0.94	0.52	0.03	0.03	0.10

* Percentiles. ** Comparison by Mann-Whitney U test.

## Supplementary Materials

The following are available online at www.mdpi.com/2072-6643/9/5/486/s1, Figure S1: Infant ASQ total score (mean ± 2 SD) at age 6 months in relation to maternal serum selenium levels in pregnancy week 36, Figure S2: Percentage of infants with infection (*n* = 19) and without infection (*n* = 88) during the first 6 weeks of life in relation to maternal serum selenium levels (tertiles) in pregnancy week 36 and 6 weeks postpartum.

## References

[1] Rayman M.P. (2000). The importance of selenium to human health. Lancet.

[2] Spallholz J.E., Boylan L.M., Larsen H.S. (1990). Advances in understanding selenium’s role in the immune system. Ann. N. Y. Acad. Sci..

[3] Kiremidjian-Schumacher L., Roy M., Wishe H.I., Cohen M.W., Stotzky G. (1994). Supplementation with selenium and human immune cell functions. II. Effect on cytotoxic lymphocytes and natural killer cells. Biol. Trace Elem. Res..

[4] Guvenc H., Karatas F., Guvenc M., Kunc S., Aygun A.D., Bektas S. (1995). Low levels of selenium in mothers and their newborns in pregnancies with a neural tube defect. Pediatrics.

[5] Skroder H.M., Hamadani J.D., Tofail F., Persson L.A., Vahter M.E., Kippler M.J. (2015). Selenium status in pregnancy influences children’s cognitive function at 1.5 years of age. Clin. Nutr..

[6] Polanska K., Krol A., Sobala W., Gromadzinska J., Brodzka R., Calamandrei G., Chiarotti F., Wasowicz W., Hanke W. (2016). Selenium status during pregnancy and child psychomotor development-Polish Mother and Child Cohort study. Pediatr. Res..

[7] Costantine M.M. (2014). Physiologic and pharmacokinetic changes in pregnancy. Front. Pharmacol..

[8] Hansen S., Nieboer E., Sandanger T.M., Wilsgaard T., Thomassen Y., Veyhe A.S., Odland J.Ø. (2011). Changes in maternal blood concentrations of selected essential and toxic elements during and after pregnancy. J. Environ. Monit..

[9] Izquierdo Alvarez S., Castanon S.G., Ruata M.L., Aragues E.F., Terraz P.B., Irazabal Y.G., González E.G., Rodríquez B.G. (2007). Updating of normal levels of copper, zinc and selenium in serum of pregnant women. J. Trace Elem. Med. Biol..

[10] Dorea J.G. (2002). Selenium and breast-feeding. Br. J. Nutr..

[11] Muntau A.C., Streiter M., Kappler M., Roschinger W., Schmid I., Rehnert A., Schramel P., Roscher A.A. (2002). Age-related reference values for serum selenium concentrations in infants and children. Clin. Chem..

[12] Jacobson B.E., Lockitch G. (1988). Direct determination of selenium in serum by graphite-furnace atomic absorption spectrometry with deuterium background correction and a reduced palladium modifier: age-specific reference ranges. Clin. Chem..

[13] Rossipal E., Tiran B. (1995). Selenium and glutathione peroxidase levels in healthy infants and children in Austria and the influence of nutrition regimens on these levels. Nutrition.

[14] Birgisdottir B.E., Knutsen H.K., Haugen M., Gjelstad I.M., Jenssen M.T., Ellingsen D.G., Thomassen Y., Alexander J., Meltzer H.M., Brantsaeter A.L. (2013). Essential and toxic element concentrations in blood and urine and their associations with diet: results from a Norwegian population study including high-consumers of seafood and game. Sci. Total Environ..

[15] Gao D., He Z., Wu J., Ma Q., Song H., Mei L., Wu Y. (1998). Long-term results of combined splenorenal shunt and porta-azygos devascularization in patients with portal hypertension. Zhonghua Wai Ke Za Zhi.

[16] Schonhaut L., Armijo I., Schonstedt M., Alvarez J., Cordero M. (2013). Validity of the ages and stages questionnaires in term and preterm infants. Pediatrics.

[17] Bolann B.J., Distante S., Morkrid L., Ulvik R.J. (2015). Bloodletting therapy in hemochromatosis: Does it affect trace element homeostasis?. J. Trace Elem. Med. Biol..

[18] Matos C., Moutinho C., Almeida C., Guerra A., Balcao V. (2014). Trace element compositional changes in human milk during the first four months of lactation. Int. J. Food Sci. Nutr..

[19] Lung F.W., Shu B.C., Chiang T.L., Lin S.J. (2009). Twin-singleton influence on infant development: A national birth cohort study. Child Care Health Dev..

[20] Hurst R., Armah C.N., Dainty J.R., Hart D.J., Teucher B., Goldson A.J., Broadlev M.R., Motley A.K., Fairweather-Tait S.J. (2010). Establishing optimal selenium status: results of a randomized, double-blind, placebo-controlled trial. Am. J. Clin. Nutr..

[21] Niskar A.S., Paschal D.C., Kieszak S.M., Flegal K.M., Bowman B., Gunter E.W., Pirkle J.L., Rubin C., Sampson E.J., McGeehin M. (2003). Serum selenium levels in the US population: Third National Health and Nutrition Examination Survey, 1988–1994. Biol. Trace Elem. Res..

[22] Kipp A.P., Strohm D., Brigelius-Flohe R., Schomburg L., Bechthold A., Leschik-Bonnet E., Heseker H. (2015). Revised reference values for selenium intake. J. Trace Elem. Med. Biol..

[23] Trafikowska U., Zachara B.A., Wiacek M., Sobkowiak E., Czerwionka-Szaflarska M. (1996). Selenium supply and glutathione peroxidase activity in breastfed Polish infants. Acta Paediatr..

[24] Monsen A.L., Refsum H., Markestad T., Ueland P.M. (2003). Cobalamin status and its biochemical markers methylmalonic acid and homocysteine in different age groups from 4 days to 19 years. Clin Chem..

[25] Greibe E., Lildballe D.L., Streym S., Vestergaard P., Rejnmark L., Mosekilde L., Nexo E. (2013). Cobalamin and haptocorrin in human milk and cobalamin-related variables in mother and child: a 9-mo longitudinal study. Am. J. Clin Nutr..

[26] Lozoff B., Beard J., Connor J., Barbara F., Georgieff M., Schallert T. (2006). Long-lasting neural and behavioral effects of iron deficiency in infancy. Nutr. Rev..

[27] Alvik A., Groholt B. (2011). Examination of the cut-off scores determined by the Ages and Stages Questionnaire in a population-based sample of 6 month-old Norwegian infants. BMC Pediatr..

[28] Watanabe C., Satoh H. (1994). Brain selenium status and behavioral development in selenium-deficient preweanling mice. Physiol. Behav..

[29] Kupka R., Mugusi F., Aboud S., Msamanga G.I., Finkelstein J.L., Spiegelman D., Fawzi W.W. (2008). Randomized, double-blind, placebo-controlled trial of selenium supplements among HIV-infected pregnant women in Tanzania: effects on maternal and child outcomes. Am. J. Clin Nutr..

[30] Stiles J., Jernigan T.L. (2010). The basics of brain development. Neuropsychol. Rev..

[31] MRC Vitamin Study Research Group (1991). Prevention of neural tube defects: results of the Medical Research Council Vitamin Study. Lancet.

[32] Kiremidjian-Schumacher L., Roy M., Wishe H.I., Cohen M.W., Stotzky G. (1992). Regulation of cellular immune responses by selenium. Biol. Trace Elem. Res..

[33] Heineman K.R., Hadders-Algra M. (2008). Evaluation of neuromotor function in infancy—A systematic review of available methods. J. Dev. Behav. Pediatr..

