# Supplementary material for: Impact of Maternal Selenium Status on Infant Outcome during the First 6 Months of Life
Source: Nutrients. 2017 May 11;9(5):486. doi: 10.3390/nu9050486 (PMC5452216; doi:10.3390/nu9050486)
Supplement: Supplementary file 1 [file nutrients-09-00486-s001.pdf]

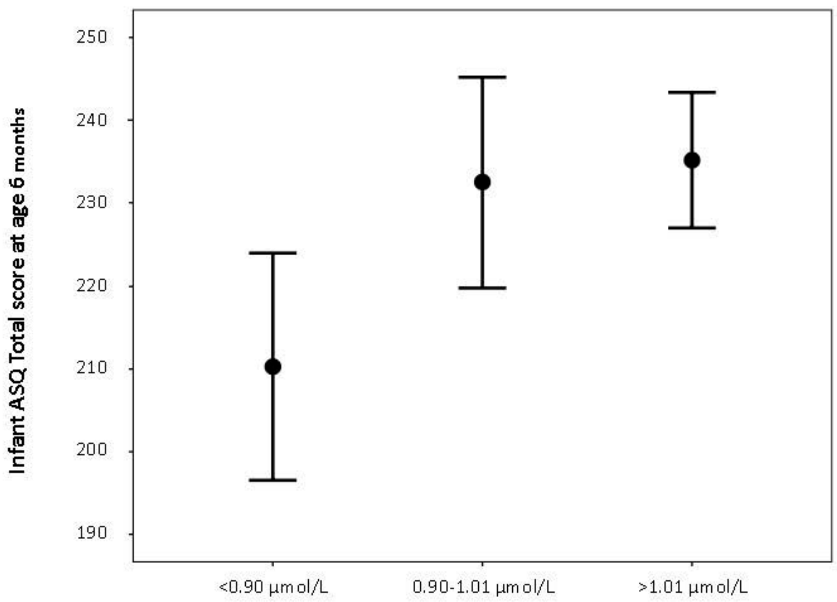

Figure S1. Infant ASQ total score (mean ± 2 SD) at age 6 months in relation to maternal serum selenium levels in pregnancy week 36.

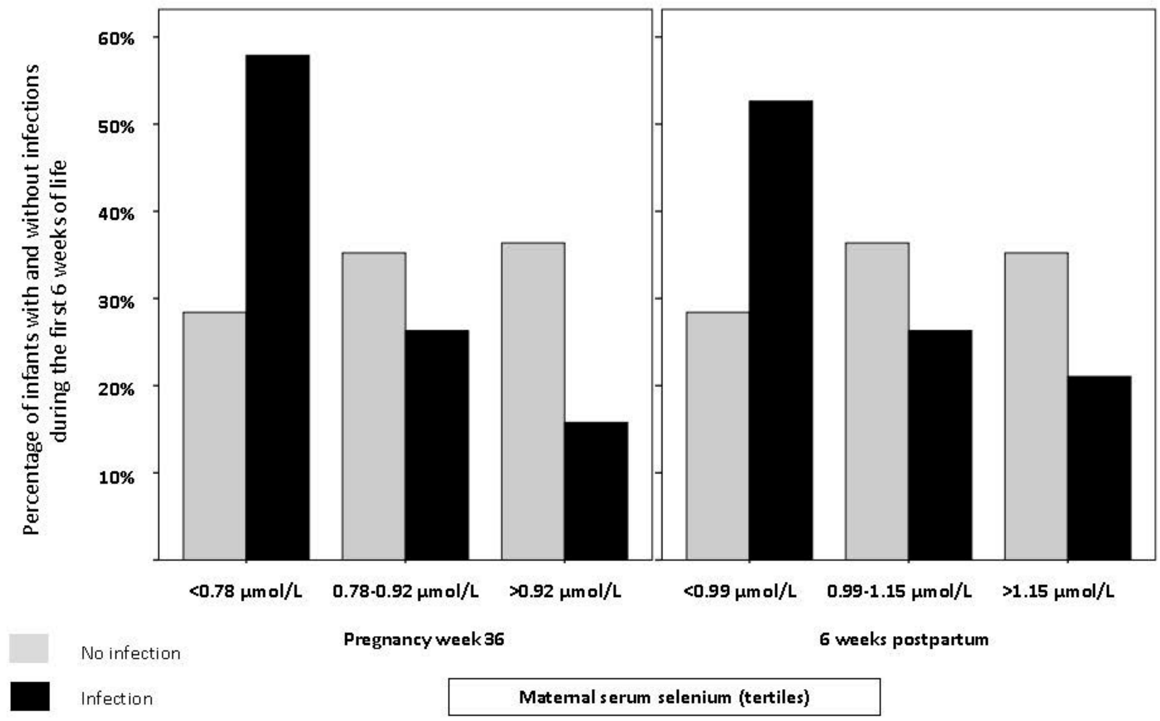

Figure S2. Percentage of infants with infection (n = 19) and without infection (n = 88) during the first 6 weeks of life in relation to maternal serum selenium levels (tertiles) in pregnancy week 36 and 6 weeks postpartum.
